# “Those Who Would Have Drifted Off Somewhere…” promoting social advancement with new learning cultures in retail

**DOI:** 10.1177/14779714231170755

**Published:** 2023-04-20

**Authors:** Patric Raemy, Lona Widmer, Antje Barabasch

**Affiliations:** 30862 University of Fribourg, Department of Communication and Media Research, Fribourg, Switzerland; 197809 Swiss Federal University for Vocational Education and Training SFUVET, Research and Development Division, Zollikofen, Switzerland

**Keywords:** social advancement, social role, learning culture, workplace training, retail

## Abstract

This study’s goal was to explore how new learning cultures might capture the many new needs of the retail industry sector and how apprentices can be trained for future work. Due to the inductive nature of this study, we found a rather implicit but important role of vocational education and training in retail that promotes the social advancement of apprentices with diverse social backgrounds. The narratives of 45 interviews with various actors involved in workplace training in one of the largest Swiss retail company revealed (1) how low-threshold and equitable access to training enables participatory engagement and self-determined success, (2) how trainers identify with social roles that are not explicitly required but are relevant for apprentices, and (3) how apprentices are given many opportunities during and after training to increase their social status. The study also revealed hindering factors such as the time and effort for the implementation of a new learning culture that affects all actors involved in workplace training. Second, are resistant negative social reproduction cycles and the heterogeneity of social backgrounds among the apprentices. Bridging this gap is simultaneously a goal and challenge for workplace training.

Retail is the second largest industry sector in Switzerland and one of the largest actors in training apprentices. Every year, a comparable high number^1^ of young people in the Swiss retail sector graduate with a Swiss Federal Certificate or Diploma of Vocational Education and Training. Many apprentices with the lowest level degree of Swiss compulsory school (or even without any school degree at all) work in the retail sector and the proportion of socially disadvantaged groups, especially women with a migration background, is high (Büchler et al., 2017). Hence, the inclusion of various groups into the (retail) labor market could be seen as an example of the success story of the Swiss dual education system (Backes-Gellner & Pfister, 2019). However, despite its reputation, the Swiss dual education system also comes with some shortcomings. For example, VET programs in retail are not the first choice for many young people and work in retail suffers from low prestige (Duemmler & Caprani, 2017). Despite its relevance as a training actor, only few graduates of compulsory school show interest to begin an apprenticeship in the retail sector.^2^ Next to the challenge of its low prestige are industrial and digital transformation in recent years that further challenge VET in the retail sector. This affected operational processes to adapt to digital transformation which led work in retail to become increasingly complex and demanding (BAK Economics, 2022).

In this study, we understand social advancement as the result of individual human development, social mobility, and social inclusion of previously underprivileged social groups in society (e.g., Blau, 1960; Ebert, 2016; Kuhlmann et al., 2018; Neelsen, 1975). Our intent is thus to explore how new learning cultures might capture the many new needs of the retail industry sector, how apprentices can be trained for future work and how their social advancement can be promoted. The Swiss retail sector promotes the inclusion of various people in the labor market, regardless of their socioeconomic status and background (BAK Economics, 2022). In this lens, VET in retail, might be a chance for social advancement of present and future employees in the retail sector, however, as previously mentioned, there might be relevant obstacles too. Hence, our endeavor in this study is to explore how a new learning culture, as a reaction of the aforementioned social and technological changes, might affect the promotion of social advancement of apprentices. The initial goal for the present case study was thus to explore the perception and implementation of a new learning culture in one of the largest retail companies in Switzerland. As part of our study’s inductive nature, the research focus shifted on how the sector-specific changes affect the renewal of VET and its orientation towards young people with different preconditions. The research goal of the study at hand is thus to investigate the social role of vocational education in retail, its potential integration function and possible opportunities for social advancement. First, we shed light on the role of VET for Swiss society with a focus on VET in retail. Second, we discuss how technological and societal changes evoke new learning cultures in the retail industry. Third, after explaining the underlying methods and sample of our study, we begin to inductively explore how the new learning culture might promote social advancement in our case study and what hindering factors might need to be considered.

The paper demonstrates (1) how the company in the case study provides its apprentices with low-threshold and equitable access to training that enables participatory engagement and self-determined success, (2) how vocational educators identify with social roles that are not explicitly required but are relevant for apprentices, and (3) how apprentices, from diverse backgrounds, are given many opportunities during and after training to increase their social status and improve their social integration. The study also shows (4) that innovative, attractive VET is possible not only in prestigious industries that recruit the most talented apprentices (e.g., the IT sector) but also in low-prestige occupations such as retail, where people with various social status and backgrounds are included in work and society.

## The social function of VET for Swiss society

The concept of social advancement is closely related to social inclusion and justice. Such terms basically indicate the need of treating all people (regardless of race, social origin, gender, or religion) equally in society with the same rights to participate in social processes and thus be equally included in society (see Ebert, 2016; Kuhlmann et al., 2018). For example, Wilson (2011) discussed the beneficial contribution of tertiary education on social inclusion. Other studies show that autonomy and diversity in the apprenticeship contribute to positive performance appraisals, career identification, and job satisfaction (Humphrey et al., 2007; Ng et al., 2005), foster career aspirations (Hofmann et al., 2014), and positively impact subsequent career integration (Stalder, 2012). Specifically focusing on VET, Nilsson (2010) sees a potential to promote social inclusion and advancement in workplace learning settings.

This study focuses on Switzerland, where vocational education has a long tradition and the highest percentage of students completing vocational training is found in Europe (about 70% of each cohort) (Gonon, 2007). After 9 years of compulsory education, apprentices start working in a host company in a chosen profession between the ages of 15 and 17. They receive a salary that increases over time. In addition to training in the host company, they attend a vocational school one or 2 days a week, where vocational and general education subjects are taught. Regular vocational training lasts three or 4 years and leads to a federal vocational qualification (Stalder & Nägele, 2011). In Switzerland, companies have a central function for the VET system. By training apprentices, companies secure the next generation of skilled workers and fulfil a social obligation for society (Bonoli & Schweri, 2019; Kuhn et al., 2018). In this context, especially the retail trade industry seems to play an important social role with its low-threshold entry into vocational training. Hence, the Swiss dual-education system might be a good example for the potential of education in promoting social inclusion and advancement, because it enables equitable access, participatory engagement, and self-determined success (Gidley et al., 2010; Gylfason & Zoega, 2003).

However, the function of education (including VET) also comes with obstacles: For example, Munro (1998) critically discussed education’s effect on the reproduction of the status quo in social differences. Regarding the division of labors, he criticizes education’s implicit role of sorting and ranking individuals for placement in the labor market in order to fill job vacancies with appropriate people. Some people will be trained for more important jobs and in reward, be given the highest incomes, while others will be less educated and will thus be given the least demanding jobs with low income. This is in line with Bourdieu’s field theory (e.g., Bourdieu & Wacquant, 1992), that explains how cultural experiences at school might differ to the experiences working-class children have at home. This might count as an argument for why children with socially disadvantaged backgrounds are not adequately prepared to cope at school. The result is a preservation of a social cycle affecting people from more difficult social backgrounds graduating in school with minimal qualifications, getting the least desirable jobs, and hence remaining socially disadvantaged groups. Hence, many capable students from socially disadvantaged backgrounds fail to achieve satisfactory standards in school and therefore fail to leave this negative cycle (Meighan & Siraj-Blatchford, 1997). This might be different in the Swiss context, where VET is considered to have positive effects on the Swiss economy and society (Bonoli & Schweri, 2019; Kuhn et al., 2018). However, arguments about education’s role in segregating social groups has relevance in Switzerland too. Abrassart & Wolter (2019) argue that the different school requirements of the training courses might promote a gap between the reputation of the professions and their actors.

Industrial and social developments also pose challenges to the VET sector in Switzerland. Labor market policies as well as individual career developments are in an ongoing process of change (Didier, 2022). These include technological, social, environmental, political-judicial, and economic changes (Schwab, 2016). Alongside expectations and behaviors among retailers and their customers change (Schwab, 2016; Swiss Retail Trade Association (BDS/FCS), see https://www.bds-fcs.ch) and transversal competences become more important. New VET curricula with a greater emphasis on the development of transversal competencies should prepare individuals better for lifelong learning, ease their access to further training and thus opportunities for social advancement (Gidley et al., 2010).

## How VET in retail adapts to social and technological changes

A major driver for change and innovation is digitalization, which in the retail industry brought online retailing to a rise, while stationary retailing declined (Rudolph & Kralle, 2020). Resulting new skill demands include a fundamental willingness to learn, to be flexible, critical thinking and the associated questioning of the status quo, a willingness to interact with others, and a variety of digital skills (IFH, 2016). Such transversal skills need to be acquired in a specific context but can be used in other contexts as well (Scharnhorst & Kaiser, 2018). This has implications for the design of workplace learning in the retail industry and consequently changes traditional ways of teaching and learning. New convictions as to how these skills can be delivered lead to a change in the existing learning culture in the sector, this is a change of attitudes, values, and beliefs as to how teaching and learning should be facilitated.

Our case study refers to the retail sector in Switzerland, where a comprehensive revision of curricula is currently taking place. Against the backdrop of the many changes in retail and the new needs and expectations in the industry, the Swiss Retail Trade Association (BDS/FCS, see https://www.bds-fcs.ch) has reformed its vocational training program based on the following assumptions: First, social changes are leading to new customer needs and expectations. Among other things, this increases the importance of customer service expertise and the importance of a good in-store shopping experience. Shopping is being promoted as a fun factor. Secondly, there are technological changes that increase a positive attitude towards new technologies, ICT skills, data protection knowledge as well as communication and sales psychology skills. They also lead to the emergence of new ways of organizing work that require employees to be more flexible in terms of location, time, and content. Third, sustainable development goals include the transfer of know-how about product and manufacturing information from retailers to customers. Finally, economic changes and on-demand sales has increased that require new skills in rapidly managing buys and sales online and improve the service across the sales line. As a consequence, a new curriculum has been developed for commercial apprenticeships, emphasizing the development of action competence, decision-making competence and responsibly. Action competence is understood as the ability to carry out professional tasks on one’s own initiative, in a goal-oriented, professional, and flexible manner (SBFI, 2022).

Retail companies need to interpret and implement the reformed vocational training program and implement it in their workplace learning. This leads to emerging learning concepts and learning cultures in the companies (Barabasch et al., 2019; Barabasch & Keller, 2020). In our research project, we examined how new work situations, competencies, values, and norms are translated into workplace learning. Like previous studies of learning cultures in companies (e.g., Johnston & Hawke, 2002; Sonntag et al., 2004), we explored new approaches to workplace learning and teaching, as well as how training concepts are perceived, negotiated, and implemented. Our previous case studies, focused on the research of learning cultures in a large Swiss telecommunication company, in the public transport sector, and at the Swiss Post (Barabasch et al., 2019; Barabasch & Keller, 2020; Keller & Barabasch, 2020). New learning cultures are phenomena that emerge from societal upheavals and challenges (Dohmen, 1996) and can thus inform the innovative capacity of VET. This points on the relevance of the connection between learning cultures and changes in society. Hence, explorations on new learning cultures in the context of workplace learning help to understand how education could promote social advancement of apprentices. The study can thus shed light on the value placed on learning, skills development, employee development, and their capacity for innovation within a company (Sonntag et al., 2004).

Scholars previously discussed the potential of new learning cultures in promoting opportunities for professional advancement and career prospects especially for socially underprivileged apprentices (DIPF, 2001; Niemeyer, 2003). VET is considered to play an important role of social integration and social advancement (Blau, 1960; Gidley et al., 2010). However, it is not yet clear whether the new industrial and social changes might be beneficial or hindering for the process of social advancement—especially in the retail sector. On the one hand, the training in retail could become more attractive because of the new focus on the development of transversal competences. This could increase the prestige of the profession and its training and attract apprentices with various school and social backgrounds. Hence, in an optimistic lens, new learning cultures might foster the inclusion of different social groups in retail. On the other hand, new learning cultures might lead to a more demanding and complex training—maybe too demanding and complex for apprentice with disadvantaged social backgrounds. Hence, although much is known about changes and new expectations in the retail sector, we know little about *the perception of apprentices and actors involved in workplace learning and how new learning cultures can promote social advancement in specific company contexts.* This case study with one of Switzerland’s largest retail companies explored how different actors involved in VET across different levels of the hierarchy perceive, negotiate, and actively shape new learning cultures, including new norms and values for work and training.

## Promoting social advancement in retail’s VET: A case study

For our case study, we chose a company whose social role is particularly relevant in Switzerland. It was founded in 1864 as a Swiss consumer association with a cooperative organization and is now one of the largest retail companies in Switzerland, an important employer and relevant training institution for many different professions. The company also publishes the largest membership weekly newspaper in Switzerland with a reach of 3.2 million readers (see WEMF Study, 2021), highlighting the company’s reach, relevance, and acceptance in society. Internal documents indicate the importance of promoting young talents and the low-threshold entry into vocational training with a variety of further training and career opportunities within the company. Basic training in the retail section is considered as a foundation for employee development and to grant next generation of professionals and future managers. The company offers versatile, practice-oriented three- or four-year basic training courses leading to a Swiss Federal Certificate of Proficiency (SFCP), as well as two-year attestation training courses (ATC). According to the company’s new training program, all apprentices need to complete a basic apprenticeship year before moving on to a one or two-year (SFCP or ATC) in-depth phase. The vocational baccalaureate is to be promoted during and after the basic apprenticeship. Further, basic education for adults will also be promoted (e.g., lateral entrants). Personal responsibility and initiative are emphasized as central values in vocational education. This is to be systematically promoted during the basic apprenticeship, among other things like stages, training sequences, and project assignments. Apprentices with above-average performance are offered further employment. High performance is to be specifically recognized, promoted, and further developed. The company in our case study also states that it is actively involved in developing new professions at the federal and industry level. The quality of training is to be ensured by reviewing training opportunities on site.

In order to investigate how new learning cultures and concepts promote social inclusion in vocational education and training, semi-structured interviews were conducted between March and July 2021 with a total of 45 apprentices, vocational trainers, regional managers, and members of the management, in both French-speaking and German-speaking regions of Switzerland. Our case study’s sample of actors involved in workplace learning reflects the new training reform of the company. Furthermore, in the sample, we paid attention to a distribution that was as representative as possible for the German- and French-speaking part of Switzerland and for the company’s different vocational training sectors in retail. The company’s head managers for VET, supported us in the recruiting process. In the interviews, we specifically asked the actors involved in workplace learning to interpret (1) the role of VET for Swiss society, (2) specific programs to promote apprentices’ professional skills and competences, (3) the promotion of talents and the support of apprentices with learning difficulties, and finally (4) the role of the trainers in supporting apprentices according to the company’s new learning culture. The narratives were coded in a qualitative content analysis according to Kuckartz (2016) and further elaborated in an iterative process through further literature review.

The narratives show that the implementation of the new training concept on the one hand increases the attractiveness of the training and the profession. There are three themes that indicate an optimistic lens on how the new learning culture might promote social advancement for apprentices: First, apprentices can take responsibility, which is promoted through addressing individual interests and action-oriented learning. Second, individual competencies are promoted through flexible training paths. Third, there is low-threshold access into the apprenticeship and thus into a community of shared experiences where more experienced employees act as role models for the apprentices. Thus, on the one hand, vocational training in the retail trade can be seen as a safety net that facilitates the transition from school to the world of work. But on the other hand, important obstacles should be considered too. The narrations revealed three themes that should be considered as hindering factors for social advancement: First, the implementation and adaptation to a new learning culture needs time and effort of all actors involved in workplace training. Second, the narrations revealed resistant negative social reproduction cycles that need to be considered when implementing new learning cultures. Finally, there is a heterogeneity of social backgrounds among the apprentices which is a challenge for workplace training in retail. Hence, our study sheds new light on the discussion about workplace learning as a problem solver and a problem creator discussed in previous studies (e.g., Di Stasio, 2017). In the following paragraphs, we first present the optimistic perspectives before discussing hindering factors for apprentices’ social advancement.

### Promoting responsibility through action orientation

One of the company’s major goals is to promote apprentices from a wide range of social contexts to become responsible employees for increasingly complex tasks. This requires a fundamental change in the national vocational training program, as a head manager of the company’s national VET explains:

*It’s not a problem to find people to perform simple tasks. But finding good people who are able to run a business—that’s not easy. Because we need 1000 people to run retail stores. We have to train them ourselves. For the more complex tasks, we don’t need people who just obey and execute things, but people who can identify and solve various problems and tasks.*

Another person from the management of the national VET program narrated that the company invested more resources and money in the training of apprentices, especially for improving the first year of the apprenticeship to provide a solid foundation for subsequent apprenticeship years. This resulted in so called “basic apprenticeships” and “basic sales outlets,” where each year, twelve retail-apprentices start their training together. After the so-called specialization phase starts, in the second and third apprenticeship years onwards where training becomes more defined according to individual preferences and abilities.

As discussed in the theoretical part of this study, the BDS/FCS highlighted the importance of competence-oriented practical assignments and the promotion of transversal competencies (e.g., independence, flexibility, creativity). The importance of this new orientation is also echoed by the narration of a manager who said, “we don’t want the apprentices to just know something, but to be able to do something with the knowledge. That means, that they can put it into practice.” The stronger focus on action-orientation in training is thus related to the company’s vision of future retail and training. The narration of the same person further revealed how practical assignments are being interpreted and implemented in the company’s new learning concept. Because of the rise of online trade, points of sale need to change and the goal for future retail stores is to increase shopping experience:

*[In future retail stores] the pastry chef will create products directly in front of the clients. Or you will be able to go directly into the cold storage room at the cheese counter, where a cheese-specialist will serve you. The experience is thus much stronger will specialize on customer experience. We thus need to promote apprentices’ individual needs and skills and support them with individual training programs. The apprentices should be able to decide after six months in which areas they want to specialize. […] In this way, we also want to respond more individually to the apprentices’ strengths and interests.*

Hence, this narration shows that the profession of retailers will change drastically. The new orientation on serving customer experience slightly aligns to other professions such as gastronomy, hostess, hotel industry. This might lead to a better prestige of professions in the retail sector. The narrations revealed that the new learning culture take a strong focus on apprentices’ responsibility and self-efficacy. Responsibility, self-efficacy, and agency have been previously discussed as related to individuals believes about their capabilities to produce designated levels of performance (Bandura, 2006) and as important factors for professional identity development (Raemy, 2021). The shift of the learning culture’s focus towards more responsibility and autonomy for apprentices learning and working which is trained through action-oriented learning might be relevant for their social advancement. To experience responsibility and action-oriented learning might be in contrast to the (for many apprentices rather less successful) experiences in compulsory school.

### Promoting individual competencies through flexible training paths

Most apprentices in our case study are young women with low educational levels and migration backgrounds. The narrations revealed that those apprentices like their apprenticeship and even found a new start after their sometimes-troubled school time. Our case study revealed kind of a social mission the company provides for VET and Swiss society, where socially disadvantaged apprentices receive many opportunities for social advancement during their training. For example, one of the managers of the company’s national VET program says:

*Many are simply tired of school and are happy to be able to work. I was the same. I believe that we can help many people who would otherwise drift off to unemployment or social problems because they can’t find work. Or they like to work in a job, but they don’t have any specific training, so they don’t have a chance to continue their career. They were not good in school and have a low social background. […] We have many of these people here—those who would otherwise have drifted off somewhere.*

The company not only offers a low-threshold range of retail apprenticeships but also caters to the many different backgrounds of its apprentices. Apprentices can benefit from a variety of career opportunities that are actively promoted. One apprentice tells:

*I will complete the SFCP and then continue my studies, for a specialist certificate or at a university. I would like to continue here in the company to become a general manager or later a sales manager. My superiors support me because they see that I am someone who works, that I am someone who is ambitious. They explain things to me that you don’t really learn until your second or third year and say, “Keep thinking and looking.” That motivates me because when I work with people who are motivated, it’s easier for me to keep moving forward.*

The goal of VET in this retail company is to promote the individual capacities of young people during and after their apprenticeship and to specifically develop young talents for the various areas of activity in the company. The apprentices can choose among numerous courses that are offered. An apprentice explains:

*At the moment I’m doing all the courses you have to do for the first year. Afterwards, I can choose. My supervisors told me to do all kinds of courses. I’ve really come across a good team, good supervisors, people who are really ambitious, who look far ahead.*

The company offers apprentices numerous opportunities for advancement in their training. For example, apprentices can start with the lowest level of training (the two-year attestation course) and then later, if they are suitable, switch to the more demanding training program (the four-year basic training with federal certificate of proficiency). It is thus possible to start with a low-threshold basic training and later, if educational success and motivation allows it, to change to a more advanced training or certification program during or after the VET. The narration of a first-year apprentice exemplifies this flexibility in training:

*I started the basic apprenticeship. […] I was just too lazy to look for something else at that time and was glad that I got this job. When I started the apprenticeship, I only had good grades. Then I turned to the apprenticeship supervisor, and he upgraded me to the extended apprenticeship after half a year. The grades have remained the same. I'm now already one of the better students at school.*

The narrations revealed that the flexible training paths might be a fostering factor especially for those who didn’t like or didn’t perform well in compulsory school. This difference in learning and performing between school and apprentice and might be a motivational factor and an opportunity for a fresh start.

### Low-threshold access into a community of shared experiences

The company can only enact its social mission and promote social advancement of young people when they can find their way into the company’s apprenticeship program. Hence, a low-threshold access into VET is important. Many apprentices have already started an apprenticeship and then changed into an apprenticeship of the company in our case study. For example, an apprentice who first completed a pre-apprenticeship in a foundation for young people with learning difficulties was then offered an apprenticeship by the company in the case study:

*It was the only apprenticeship I was offered. For me, it’s an opportunity to try something new. If I don’t like it, it's only two years and the first year is almost over already.*

Many of the more experienced and hierarchically higher-ranked employees have completed workplace training in this company too and now serve as role models for the apprentices. Hence, many of the persons in our case study made their career in this company. Their careers lead from compulsory school through an apprenticeship in retail to a management position in retail business that can include up to hundreds of employees. Therefore, they know the effort it takes to advance personally and professionally—and eventually their social status. They are aware of the numerous challenges that young people have to deal with, have an understanding for their concerns and are available as competent contact persons and advisors. The company’s goal, then, is not only to select and promote talents but also to support those who have difficulties in learning. There is a fundamental belief that everyone in the company is needed and that commitment to all apprentices pays off. The story of a retail manager is exemplary:

*I was not good at school. After I got an apprenticeship at the company, I was very committed. I wanted to show everyone that I could work well. My training was not easy. In my first job after the apprenticeship, I had a boss who motivated me and promoted me to the assistant store manager. He also hired an apprentice who grew up in a home for people with special needs. I had responsibility to care about him. He was a rather weak apprentice, but we learned a lot from each other. For me, it has always been important to have one, two or three “weaker” apprentices. Usually, they turned out to be very good professionals—that’s what I've been doing all these years.*

The narrations revealed the relevance of role models in career decisions which is in line with findings of previous studies (e.g., Neuenschwander et al., 2018). Hence, especially in retail, the influence of more experienced workers on apprentices learning and career paths might promote social advancement in a sector with rather low prestige and many young people with different social backgrounds. This could be seen as a social mission for VET in retail and for Swiss society in general.

The narrative illustrates the low-threshold nature of the training, where progressively higher degrees can be earned. The case study shows where this system works and adapts to new requirements and new generations. Through competence-oriented practical assignments, transversal competences can be specifically promoted. Individual needs of the apprentices are directly taken into account through individual and self-selected consolidation phases. Further the personal support by the vocational trainers and their role as advisors shows that apprentices from all social backgrounds are integrated and supported. This social function of VET is often implicitly perceived by the actors. Our study revealed that most trainers are not explicitly aware of their role of promoting apprentices’ social advancement. Mostly they rather see it as a positive side-effect of vocational training.

### A change needs time

The narratives also reveal limits and obstacles of social advancement in retail. The change in learning cultures is not easy to initiate and implement. After all, a new learning culture needs to be accepted and implemented by all stakeholders involved in workplace training in a company. A change in learning cultures is a process, that needs time and effort from all actors involved in workplace training. Actors perceive new expectations and changes different, which leads to various forms of enactment of the new learning culture’s initial ideas (Raemy & Barabasch, 2022a). This fact influences the apprenticeship significantly. A person from the management of national vocational training program explains:

*The apprentice’s supervisors often still work according to the ideas of the old training concept and do not want to adapt to the new ideas. We try to convince them by showing them the advantages of the new training concept. The resistance is decreasing, but we are aware that it will take time until everything really works. A project like this needs support, and we provide it.*

The difficulties in adapting to changes and new expectations can be seen in some trainers’ perceptions on the new focus of action-oriented learning. Another person from the management of national vocational training program explains:

*The goal for VET trainers should not be to set deadlines for apprentice’s practice assignments. Instead, they should ask apprentices about their progress and potential difficulties. […] This is a huge role change. It certainly needs time till all our people will understand it [the idea of the new learning culture].*

This process of perception, negotiation, normalization, and enactment of changes and new expectations need to be considered when implementing a new learning culture in workplace learning (Raemy & Barabasch, 2022a). This also means a shift in many trainers’ professional role that needs to be negotiated and adapted (Raemy & Barabasch, 2022a). Hence, both needs time and effort, the implementation of a new learning culture as well as social advancement of disadvantaged groups.

### Resistant negative social reproduction cycle

Another theme is how external factors or inert structures might affect new learning cultures potentials for social advancement. Bourdieu’s (1977) theory of reproduction of social cycles might confront some potentials of new learning cultures because the social backgrounds of apprentices might play a central role in the choice of their career and learning paths (Kupfer, 2011). The narratives showed that teachers’ and parents’ ideas about retail are passed on to apprentice. A narrative of a person responsible for apprentice support is exemplifying:

*I think that [the retail career image] is sometimes influenced in students by parents and teachers. When we have vocational events on site, we are in contact with teachers and so on. We notice that the interest of the young people is not there, and when you then talk to the teachers, you notice that they don’t necessarily want their students to go into retail trade or logistics but rather to go into another field. There are also teachers who think that their students wouldn’t be interested in retail anyway. That’s what I mean when I say that it’s controlled by teachers and parents, that the retail trade is not necessarily the most attractive.*

The reproduction of structures and opinions seems to be a hurdle for the implementation of innovative ideas, as newly arriving apprentices are partly unsettled and less motivated due to the image of the profession and industry. The narration of an apprentice about her decision to become a retailer is exemplary for disputes about career choice between parents and their children:

*My mother totally panicked and wanted me to go to the office or something. She said, “You won’t get anywhere like that. Even I can do that. Everyone can just stand in the store and fill shelves. “ My father told me to do what makes me happy. That already supported me. I know that my parents wanted me to do something, in their eyes, “better.” […] But it wouldn’t have made me happy to sit in an office all day.*

Parental beliefs about children’s career decisions are related to their own experiences in vocational education, the exchange of information with peers, and the media image of an industry. Parents influence their children with both their level of knowledge and their attitudes, values, and beliefs (Pruisken, 2018; Steinmann, Maier & Lohaus, 2018). Teacher discourse also has a significant impact on students’ decisions regarding career choices (Fuller & Unwin, 2009). Narratives likewise highlight the influence of social background on concrete educational decisions of apprentices (social origin effects according to Boudon, 1974; Becker, 2004). These tendencies complicate to change culture within an organization. At the same time, the narratives show that role models who do not confirm existing images can contribute significantly to reducing the influence of social origin.

### The heterogeneity among the apprentices as a challenge

Furthermore, it is always a challenge for education and training to address the increasingly complex industrial needs without overtaxing apprentices. This is especially true for the establishment of new learning cultures. Accordingly, the company of our case study is challenged to offer low-threshold vocational training also for apprentices with learning difficulties. The new learning concept and the new learning culture aim at developing independent working and learning as well as self-initiative. With less guidance and more accompaniment and coaching, the independence of the apprentices is promoted; at the same time, softened structures and less guidance could be challenging especially for apprentices with learning difficulties. In addition, there is a risk that, as a result of increased performance requirements, the entry threshold for professions in the retail trade will become higher and the requirements more complex. A person from apprentice support says:

*I don’t think that the requirements have become fundamentally higher. But the problem is sometimes the quality of the candidates who come to us. They bring less knowledge and know-how to be able to do the apprenticeship. Among migrants, maybe understanding is a challenge. It doesn’t mean they can’t do it, but it takes them longer to do what we ask them to do. […] Another problem is that our profession is often considered as a second or third choice for many apprentices. Good apprentices who could be taken as retail specialists may not necessarily decide to go into retail first, but instead do some kind of school-based training or go more in the direction of commercial training. I don’t think that our apprentices are worse, but they are perhaps sometimes more challenged with school. That’s why I think that the hurdles for them are correspondingly higher.*

A training concept that promotes independent learning and working could be challenging especially for those with learning difficulties. For example, there is a risk that those with weaker learning, who need more direction and managerial structures, will lose their connection to the training. One person from management describes that it is a challenge to integrate all apprentices into the new learning culture and the new training concept:

*We are now applying a concept to 1,000 young people who started in August. Of course, it’s not right for everyone. We have young people who can get involved more quickly and therefore find it easier. We also have those who need a certain amount of support and guidance. We have young people who come from a social environment that makes the training very difficult, who can’t concentrate on the work at all because they are always carrying so many problems around with them.*

Hence, the heterogeneity among the apprentices (and actors involved in workplace training), should be considered when implementing new learning cultures in workplace learning. This is especially important regarding apprentices’ social advancement, where balancing on the fine line between addressing the complex industrial needs without overwhelming apprentices might become increasingly important.

## Conclusion

This study’s goal was to explore the perception of apprentices and actors involved in workplace learning and to examine how new learning cultures can promote social advancement in a specific company context*.* The case study revealed two main perspectives on workplace learning in retail:

First, there is a rather optimistic perspective, which describes the beneficial effects of the new learning culture on apprentices’ social advancement. The new learning culture represents a new understanding of competence development with a stronger emphasis on the development of transversal competences, such as independence, responsibility, creativity, etc. The improved training has the potential to promote social advancement. The narratives also showed, that this important function and benefit for the Swiss society, might not be very popular and the actors in our sample mostly saw social advancement rather as a positive side effect than an explicit educational goal. In addition, narrations indicated, that young, often female apprentices from different, mostly rather disadvantaged, social backgrounds are given many opportunities to increase their social status and improve their social integration during and after their training. This confirms, what we learned from theory about social advancement (Blau, 1960; Gidley et al., 2010): The retail company in the present case study promotes social inclusion and advancement of less privileged members in society by providing its apprentices with low-threshold access and flexible career paths. The narratives revealed that workplace learning in retail have the potential for socially disadvantaged apprentices to leave a problematic environment (family, peers, and school), entering a new community of people with similar experience and backgrounds, and thus might take a fresh start in a supportive educational and working environment. The new learning culture might promote their responsibility and self-efficacy and thus increases their chances for employment and career advancement. This can be best exemplified by the narrative of an apprentice who was given the opportunity to assist the store manager:

*The deputy general manager then said to me that he was giving me a certain role of management. If I had any questions, I could turn to him. But he also said that he trusted me and that I could certainly lead the team there, that I had the competence to hold the whole team together a little bit.*

Our case study shows that innovative VET is not limited to prestigious sectors (e.g., the IT sector), where the most talented apprentices can be recruited. New, innovative learning cultures can also work in low-prestige professions such as retail, where the positive educational side-effect of promoting social advancement might be stronger. The following narrative of a vocational trainer illustrates this fact:

*I care for these young people. I guide them. My youth, how I grew up wasn’t straight nor was my own school time and training. Most of the time they spend with their families, their free time, their friends—which are important for them. That’s why I can catch them also in matters not only related to work.*

The narration exemplifies the important role of trainers for their apprentices’ social development and advancement. They take on roles that are not explicitly articulated to be their professional roles—and it seems that they often enact these roles rather unconsciously, because they think it is the right thing to do. Out of a perspective of social advancement, these roles seem as relevant as other roles related to work training.

Second is the perspective on obstacles and hindering factors. The case study revealed resistant structures in workplace learning such as retails negative reputation in society. Parents’ and teachers’ perpetuation of negative connotations with retail might affect their children or students and thus further promote the negative social reproduction cycle. Some narrations of the managers indicated a fear of promoting “a catch basin” due to the low threshold entry into the training that attract weaker apprentices. This might be seen in conflict with economic goals of VET and managers thus not explicitly and publicly promote the social function of VET in retail. Our case study further indicated the challenge of new learning cultures of balancing on the fine line between addressing the complex industrial needs without overtaxing apprentices. Finally, new learning cultures come with new expectations and changes that actors need to adapt to. Hence, implementing a new learning culture should be seen as a process of perception, negotiation, normalization, and enactment of changes (Raemy & Barabasch, 2022a) and as a shift in many trainers’ professional role (Raemy & Barabasch, 2022a).

Our study shows that structures and actions are in a reciprocal relationship. The new innovative ideas that should lead to new learning and actions ought to be communicated in a consensus-building process in order to evoke changes in structures and ways of working. Changes occur more easily when the change within VET is of a global nature, that is, when it also affects other retail companies. To this end, it helps to locate communication about innovative approaches publicly. In this way, change can be accelerated and forced within the organization as a result of the externalization potential. More research is needed to further explore how retail’s promotion of social advancement might affect public communication and hence lead to a better reputation of its profession and training.

Our case study’s focus is on the Swiss retail industry. Future studies might explore other country and educational contexts. It might be interesting to explore how trainers and teachers in other contexts might promote social advancement of socially disadvantaged learners. Further case studies could serve as examples of good practice to ensure that companies continue to be aware of this important mission, to keep on improving it, and enact their social function.

## Notes

1. The exact numbers of employment and graduations are listed at https://www.bfs.admin.ch

2. See for example, https://www.cockpit.gfsbern.ch
